# Is fenofibrate a cost-saving treatment for middle-aged individuals with type 2 diabetes? A South African private-sector perspective

**DOI:** 10.5830/cvja-2010-001

**Published:** 2010-02

**Authors:** Francois Wessels

**Affiliations:** Outcomes Consultants, Pretoria, South Africa

**Keywords:** type 2 diabetes, pharmaco-economics, fibrate therapy, cardiovascular disease

## Abstract

**Introduction:**

This project was based on the FIELD trial.^1^ It is a localisation of the study by Carrington and Stewart.^2^ The aim of the original study was to determine the impact of fenofibrate therapy on healthcare costs of middle-aged patients with type 2 diabetes at high risk of future cardiovascular events.

**Methods:**

The methodology used in the Carrington article^2^ was adopted for this study. The clinical foundation for the analysis was derived from the findings of the FIELD study.^1^

All costs were sourced from electronic databases obtained from private-sector South African funders of healthcare. Event costs for the cardiovascular events were determined and added to the treatment costs for the individual treatment arms. The cost saving was determined as the difference between the event costs saved and the additional treatment costs associated with fenofibrate treatment. All costs were reported as 2008 ZAR and a discount rate of 10% was used. The study adopted a South African private-sector funder perspective.

**Results:**

If the same approach is followed as in the Carrington and Stewart study,^2^ a cost saving of 18% results. This is the difference between the total costs associated with the placebo and fenofibrate arms, respectively (R3 480 471 compared to R2 858 598 per 1 000 patient years for the placebo and fenofibrate arms, respectively). The total costs were determined as the sum of associated event costs and treatment costs for each of the comparators.

**Conclusions:**

Based on this exploratory analysis, it seems that Lipanthyl® treatment in middle-aged patients resulted in a cost saving due to the prevention of cardiovascular events when it was used in the treatment of type 2 diabetics, as in the FIELD study. It should therefore be considered to be cost effective, even when just the cardiovascular risk reduction effect is considered.

## Summary

The Lipanthyl Intervention and Event Lowering in Diabetes (FIELD) study was a multinational, randomised trial of 9 795 patients aged 50 to 75 years with type 2 diabetes mellitus. Eligible patients were randomly assigned to receive Lipanthyl 200 mg/day (*n* = 4 895) or matching placebo (*n* = 4 900).

At each clinic visit, information concerning laser treatment for diabetic retinopathy – a prespecified tertiary endpoint of the main study – was gathered. Adjudication by ophthalmologists masked to treatment allocation defined instances of laser treatment for macular oedema, proliferative retinopathy, or other eye conditions.

In a sub-study of 1 012 patients, standardised retinal photography was done and photographs were graded with Early Treatment Diabetic Retinopathy study (ETDRS) criteria to determine the cumulative incidence of diabetic retinopathy and its component lesions. Analyses were by intention to treat. This study was registered as an international standard randomised controlled trial, number ISRCTN64783481.

Based on the FIELD study,^1^ a pharmaco-economic analysis of the use of Lipanthyl in preventing cardiovascular events has been developed. This study modelled the economics of Lipanthyl use in type 2 diabetes patients, focusing on the cost-saving impact of Lipanthyl treatment in reducing the risk of cardiovascular events due to myocardial infarction (MI), stroke and the need for angiography and revascularisation procedures. According to Carrington and Stewart,^2^ type 2 diabetes is associated with increased risk for cardiovascular disease (CVD).

## Significance of this and previous studies

Fibrates are currently receiving renewed attention in South Africa. Lipanthyl has some of the strongest evidence in the class of fibrates. The recent FIELD study^3^,^4^ with its 9 795 patients positioned Lipanthyl as perhaps the strongest contender in this class.

The clinical argument has been well established by means of the evidence-based medicine supporting Lipanthyl. Not only has its efficacy been established in numerous clinical trials, but its effectiveness in the reduction of clinical events has now been proven by the FIELD study as well.

Pharmaco-economic evidence is critical in obtaining reimbursement in private healthcare in South Africa. With the FIELD study, the opportunity is now presenting itself to develop not only a compelling clinical argument but also a definitive economic or value argument to support the use of Lipanthyl as an essential component in lipid management.

With such a large study as FIELD^4^ now available, the potential of a pleiotropic effect associated with Lipanthyl will be explored. Bearing these arguments in mind, the development of economic evidence as part of the portfolio of evidence associated with Lipanthyl is not only justified, but is critical to position the molecule in the market window presenting itself at present.

In the FIELD study at five-year follow up, a similar proportion of subjects in both groups (10–11%) had discontinued therapy (FIELD study group: 2005).^1^ However, those randomised to placebo were more likely to require supplementary lipid-lowering therapy (in this case, statin therapy) compared to the fenofibrate group. There was no difference in the rate of starting other lipid-lowering treatment between the groups in those with a history of cardiac events (23 vs 14%) and those without a history at baseline (16 vs 7%). However, for those with previous CVD, the average rate was higher.

**Fig. 1.**
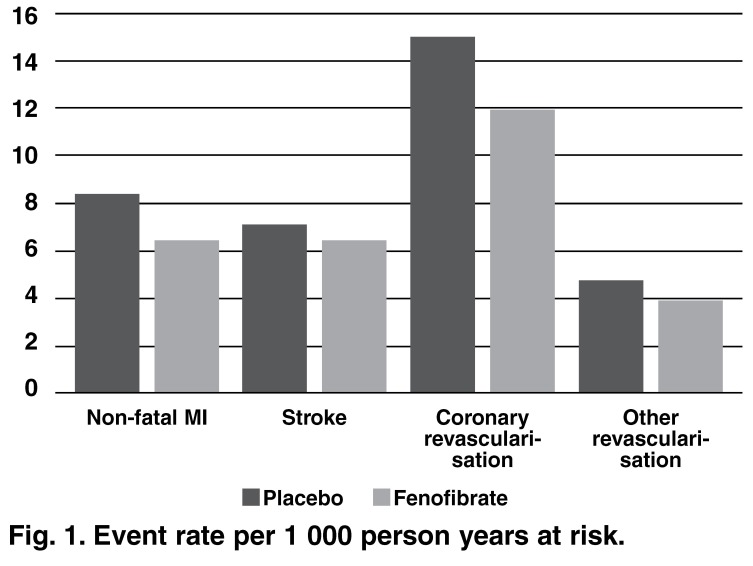
Event rate per 1 000 person years at risk.

Despite a similar all-cause mortality in the FIELD study^1^ (6.6% in the placebo group vs 7.3% in the fenofibrate group), fenofibrate was associated with (see also Fig. 1):

● 11% risk reduction for a coronary event (*p* = 0.16)
● 24% risk reduction for non-fatal MI (*p* = 0.010)
● 19% risk increase for CHD mortality (*p* = 0.22)
● 11% risk reduction for a cardiovascular disease event (*p* = 0.035)
● 21% risk reduction for coronary revascularisation (*p* = 0.003).

Although fenofibrate did not significantly reduce the risk of the primary outcome (coronary events) relative to treatment with placebo (standard treatment), it was associated with fewer non-fatal myocardial infarctions and revascularisations in this relatively high-risk group of patients.

## Study aim

This analysis has developed an economic assessment of the reduction of the risk associated with myocardial infarction, stroke and the need for angiography and revascularisation in type 2 diabetes mellitus patients. This study therefore aimed to prove that the effect of Lipanthyl in reducing the risk associated with cardiovascular disease in subjects with type 2 diabetes mellitus constituted a cost-saving intervention. In order to test this hypothesis, a cost–consequence analysis was followed up with a cost–benefit analysis.

## Methods

## Study design

The study was a localisation of the Carrington and Stewart study reported in the *International Journal of Cardiology.*^2^ The methodology used in their study was applied here as well, to ensure integrity of the findings. This included the parameters used in the study. The methodology used by Carrington and Stewart is described comprehensively in their article, but for convenience, it will be summarised here, indicating which adaptations were required to localise the study.

This was a retrospective pharmaco-economic study based on cost–consequence and cost–benefit analyses. The study population consisted of patients suffering from type 2 diabetes mellitus, as in the FIELD study.^1^ Significance levels and the power of the test have been derived from the FIELD study

The total treatment cost was determined for the Lipanthyl and control groups. In the process of calculation, clinical effects and resource utilisation were analysed and reported. The incidence of all clinical events is reported as events per 1 000 person years. The clinical effects that were considered included:

● non-fatal myocardial infarctions (ICD10 codes I23 to I25)
● as in the Carrington article, it was assumed that all patients with a reported non-fatal MI underwent a coronary angiogram (CPT codes 93543/5 and 93555)
● stroke (ischaemic stroke: ICD10 codes I63–I64 and haemorrhagic stroke: ICD10 codes I61–I62)
● coronary revascularisation (limited to stents – both bare metal stents and drug-eluting stents were included at the rate in which they occurred in the database of a local healthcare funder); the fact that coronary artery bypass grafts were not included may be considered to understate the event costs and would therefore constitute a conservative estimation of costs in this regard
● non-coronary revascularisation.

The resource effects considered consisted of:

● hospitalisation costs
● pharmacotherapy
● professional fees.

The discount rate used in this analysis was 10%, which is a conservative estimate of the 2008 R153 government bond rate. A private-sector funder perspective was used.

The active intervention used in the comparison was Lipanthyl 200 mg/day as per the FIELD study.^1^ The control group in this comparative study was placebo, which in this case refers to standard treatment. The lipid-lowering therapy introduced in this analysis was 10 mg of simvastatin at the average generic price for the South African private sector. This can be considered to be a conservative approach and adding any other lipid-lowering agents would have only increased the difference in costs associated with the two comparators. All acquisition costs are reported as retail price ex VAT.

## Analysis

The analysis was based on the intention-to-treat population from the FIELD study.^1^ The time frame for the base case analysis has been derived from the FIELD study and was five years or 60 months.

All the clinical outcomes were derived from the FIELD study. One-way sensitivity analyses on risk reduction were done to assess the robustness of clinical findings as well as assumptions relating to treatment costs and resource utilisation.

A cost-effectiveness threshold of ZAR95 000 per QALY (quality-adjusted life year) has been used. This is the equivalent of one times the South African per capita GDP, which should be considered a very conservative estimate relevant to international convention.

This was followed by a cost–benefit analysis, which compares drugs or programmes by expressing both outcomes and resource utilisation in monetary terms in a situation where, for a given level of resources, the decision maker wishes to maximise the health benefits conferred to the population of concern.

A budget impact analysis assessed the net cost impact of the intervention compared with the standard therapy for any defined target population as per the perspective of the study. In this analysis the total costs associated with each treatment arm were expressed as the sum of event costs and treatment costs.

The costs used in this analysis were obtained from three sources. These were electronic databases obtained from private-sector healthcare funders. These sources are indicated per event in Table 1. The coronary revascularisation and angiography costs were derived from a Healthcorp database, MI event costs were obtained from a Medihelp database, and stroke and other revascularisation costs were obtained from a Solution database.

**Table 1:** Adjusted Within-Trial Cost Comparison Of Fenofibrate Versus Placebo: Five-Year Follow-Up Cohort Of The Field Study

	*Event comparison/1 000 person years at risk*			*Cost comparison*		
	*Placebo*	*Fenofibrate*	*Cost per unit (ZAR)*	*Placebo*	*Fenofibrate*	*Difference*
Non-fatal MI	8.4	6.4	R 110 475**	R 927 990	R 707 040	R–220 950
Coronary angiography	8.4	6.4	R 17 298***	R 145 304	R 110 708	R–34 596
Stroke	7.1	6.4	R 54 739^#^	R 388 647	R 350 330	R–38 317
Coronary revascularisation	15.0	11.9	R 127 937***	R 1 919 048	R 1 522 445	R–396 603
Other revascularisation	4.7	3.9	R 19 734^#^	R 92 748	R 76 961	R–15 787
R–706 254 in favour of fenofibrate						
	*Treatment comparison/1 000 person years of treatment*			*Cost comparison*		
	*Placebo*	*Fenofibrate*	*Cost per annum (ZAR)*	*Placebo*	*Fenofibrate*	*Difference*
Lipid-lowering therapy	4.4	2.1	R 1 530*	R 6 734	R 3 214	R–3 520
Fenofibrate therapy	–	25.2	R 3 488M^##^	–	R 87 901	R 87 901
R 84 381 in favour of standard treatment						
			*Total cost*	*Placebo*	*Fenofibrate*	*Difference (%)*
Cost saving of R–621 873 per 1 000 patient years of treatment with fenofibrate				R 3 480 471	R 2 858 598	18%

*Simvastatin 10 mg (30); ^#^Solution data; **Medihelp data; ***Healthcorp data; ^##^Retail ex VAT

All treatment costs are SEP (single exit price) including VAT and are reported for the same exposure period as the cardiovascular events. The treatment costs are therefore expressed as cost per 1 000 person years of treatment.

A one-way sensitivity analysis was conducted using the 95% confidence intervals of the event rates. These results are reported in Tables 2 and 3.

**Table 2:** Adjusted Within-Trial Cost Comparison Of Fenofibrate Versus Placebo: Five-Year Follow-Up Cohort Of The Field Study Based On 95% CI For Observed Event Rates: Higher 95% Confidence Limits

	*Event comparison/1 000 person years at risk*			*Cost comparison*		
	*Placebo*	*Fenofibrate*	*Cost per unit (ZAR)*	*Placebo*	*Fenofibrate*	*Difference*
Non-fatal MI	8.4	5.2	R 110 475**	R 927 990	R 574 470	R–353 520
Coronary angiography	8.4	5.2	R 17 298***	R 145 304	R 89 950	R–55 354
Stroke	7.1	4.7	R 54 739^#^	R 388 647	R 257 273	R–131 374
Coronary revascularisation	15.0	10.2	R 127 937***	R 1 919 048	R 1 304 953	R–614 095
Other revascularisation	4.7	3.3	R 19 734^#^	R 92 748	R 65 121	R–27 627
R–1 181 970 in favour of fenofibrate						
	*Treatment comparison/1 000 person years of treatment*			*Cost comparison*		
	*Placebo*	*Fenofibrate*	*Cost per annum (ZAR)*	*Placebo*	*Fenofibrate*	*Difference*
Lipid-lowering therapy	4.4	2.1	R 1 530*	R 6 734	R 3 214	R–3 520
Fenofibrate therapy	–	25.2	R 3 488^##^	–	R 87 901	R 87 901
R 84 381 in favour of standard treatment						
			*Total cost*	*Placebo*	*Fenofibrate*	*Difference (%)*
Cost saving of R–1 097 589 per 1 000 patient years of treatment with fenofibrate				R 3 480 471	R 2 382 882	32%

*Simvastatin 10mg (30); ^#^Solution data; **Medihelp data; ***Healthcorp data; ^##^Retail ex VAT

**Table 3:** Adjusted Within-Trial Cost Comparison Of Fenofibrate Versus Placebo: Five-Year Follow-Up Cohort Of The Field Study Based On 95% CI For Observed Event Rates: Lower 95% Confidence Limit

	*Event comparison/1 000 person years at risk*			*Cost comparison*		
	*Placebo*	*Fenofibrate*	*Cost per unit (ZAR)*	*Placebo*	*Fenofibrate*	*Difference*
Non-fatal MI	8.4	7.9	R 110 475**	R 927 990	R 872 753	R–55 238
Coronary angiography	8.4	7.9	R 17 298***	R 145 304	R 136 655	R–8 649
Stroke	7.1	7.9	R 54 739^#^	R 388 647	R 432 438	R 43 791
Coronary revascularisation	15.0	13.9	R 127 937***	R 1 919 048	R 1 778 318	R–140 730
Other revascularisation	4.7	4.3	R 19 734^#^	R 92 748	R 84 855	R–7 893
R–168 719 in favour of fenofibrate						
	*Treatment comparison/1 000 person years of treatment*			*Cost comparison*		
	*Placebo*	*Fenofibrate*	*Cost per annum (ZAR)*	*Placebo*	*Fenofibrate*	*Difference*
Lipid-lowering therapy	4.4	2.1	R 1 530*	R 6 734	R 3 214	R–3 520
Fenofibrate therapy	–	25.2	R 3 488^##^	–	R 87 901	R 87 901
R 84 381 in favour of standard treatment						
			*Total cost*	*Placebo*	*Fenofibrate*	*Difference (%)*
Cost saving of R–84 338 per 1 000 patient years of treatment with fenofibrate				R 3 480 471	R 3 396 133	2%

*Simvastatin 10mg (30); #Solution data; **Medihelp data; ***Healthcorp data; ##Retail ex VAT

## Results

The cost-effectiveness analysis was based on the methodology described in the article by Carrington and Stewart.^2^ The results from this analysis are reported in Table 1.

As is evident from this table, fenofibrate therapy was consistently associated with a reduction in the incidence of cardiac events. Per 1 000 person years, fenofibrate therapy was therefore associated with a cost saving of R706 254 resulting from events avoided over the 1 000 person-year observation period. This amount was calculated as the product of the unit cost of an event and the number of events per 1 000 person years estimated to be associated with the respective treatment arms.

To this we then added therapy costs. Fenofibrate therapy resulted in an increase of R84 381 in pharmacotherapy costs. The sum total of the event costs and therapy costs was calculated to be R 3 480 471 in respect of the placebo arm and R 2 858 598 for the fenofibrate therapy. A resultant cost saving of R 621 873 was modelled to result from the introduction of fenofibrate treatment to the treatment of middle-aged type 2 diabetics.

The one-way sensitivity analysis (see Tables 2 and 3) indicated that the analysis was robust as far as the event-rate assumptions were concerned. Both for the upper and lower 95% limits, a cost saving associated with fenofibrate was indicated (R1.098 million and R84 000, respectively).

## Conclusion

Based on this analysis, it appeared that Lipanthyl® resulted in a cost saving due to the prevention of cardiovascular events, including myocardial infarction and stroke, when it was used in the treatment of type 2 diabetics as in the FIELD study. It should therefore be considered to be cost effective, even when just the cardiovascular risk-reduction effect is considered. The addition of fenofibrate should therefore be favourably considered for inclusion in the management regimes for type 2 diabetes.

These results were indicated to be robust to the assumptions made regarding the event rates, as was suggested by the one-way sensitivity analyses that were done.
